# Limitations in the study of restless legs syndrome and ferritin levels in older adults with dementia

**DOI:** 10.1590/1980-5764-DN-2025-0348

**Published:** 2025-12-01

**Authors:** Almas Gul, Ramsha Gul

**Affiliations:** 1 Liaquat University of Medical and Health Sciences, Faculty of Medicine and Allied Sciences, Department of Medicine, Jamshoro, Sindh, Pakistan.

 Dear Editor, I read with great interest the article titled "Restless Legs Syndrome and Ferritin Levels in Older Adults with Dementia: A Cross-Sectional Study" by Leite et al.^1^. The study provides valuable insights into the association between Willis–Ekbom disease/restless legs syndrome (WED/RLS) and iron-deficiency anemia in older adults with dementia. The finding that 15.7% of these patients had RLS and ferritin deficiency highlights an important area of clinical concern. However, I would like to address a few potential limitations that could impact the interpretation of the findings. 

 Firstly, a significant portion of the data in this study was obtained from secondary and tertiary caregivers^1^, which introduces the possibility of reporting bias. These caregivers, who may have less frequent or less intimate contact with the patients, might emphasize symptoms they perceive as more significant or socially acceptable, potentially skewing the data. Although the exclusion of primary caregivers aimed to reduce emotional bias, secondary and tertiary caregivers often have a less comprehensive understanding of the patients’ behavioral and emotional baselines. For instance, studies have shown that caregivers often report higher symptom prevalence rates compared to the patients themselves^2^, potentially leading to both under-reporting and over-reporting of symptoms. Additionally, the study relied on self-reported data from patients^1^, which is vulnerable to recall bias. Self-reports are also influenced by response style effects, such as a tendency to select extreme answers^3^, a concern particularly pronounced in individuals with cognitive impairment. Future studies could benefit from incorporating more objective measures, such as neurological assessments or polysomnography, to enhance data reliability. 

 Secondly, the study was conducted during the COVID-19 pandemic^1^, a period marked by social isolation, reduced physical activity, and disrupted routines. Older adults were particularly vulnerable to the mental health impacts of the pandemic^4^, including elevated rates of depression, anxiety, and stress. These factors are common comorbidities in dementia and could have exacerbated neuropsychiatric symptoms, potentially inflating the observed prevalence or severity of RLS symptoms. 

 Thirdly, although the study included patients aged 60 years and older^1^, it did not account for the presence of multiple chronic conditions. It is well established that older adults frequently have more than one chronic illness, and multi-morbidity may influence both the risk and presentation of dementia^5^. Chronic conditions such as diabetes^6^, cardiovascular disease, hypertension, and chronic kidney disease (CKD) have been associated with both iron deficiency and RLS^7,8^. CKD, in particular, is a well-documented cause of iron deficiency anemia^9^, and heart failure is another condition where iron deficiency is common^10^. Failing to control for these comorbidities may confound the association between low ferritin and RLS, possibly exaggerating the strength of this relationship in the dementia population. 

 In summary, while this study provides valuable initial insights, a more comprehensive research design that includes objective clinical assessments, consideration of the pandemic’s psychological impact, and thorough control for chronic comorbidities is essential for a more accurate understanding of the relationship between WED/RLS and ferritin levels in older adults with dementia. 

## Data Availability

The datasets generated and/or analyzed during the current study are available from the corresponding author upon reasonable request.

## REPLY

The authors of the article were contacted by the editorial team regarding the submission of their reply; however, they declined to respond.
